# cisPath: an R/Bioconductor package for cloud users for visualization and management of functional protein interaction networks

**DOI:** 10.1186/1752-0509-9-S1-S1

**Published:** 2015-01-21

**Authors:** Likun Wang, Luhe Yang, Zuohan Peng, Dan Lu, Yan Jin, Michael McNutt, Yuxin Yin

**Affiliations:** 1 Institute of Systems Biomedicine, Beijing Key Laboratory of Tumor Systems Biology, Peking University Health Science Center, Beijing 100191, China; 2 Department of Pathology, School of Basic Medical Sciences, Peking University Health Science Center, Beijing 100191, China

## Abstract

**Background:**

With the burgeoning development of cloud technology and services, there are an increasing number of users who prefer cloud to run their applications. All software and associated data are hosted on the cloud, allowing users to access them via a web browser from any computer, anywhere. This paper presents cisPath, an R/Bioconductor package deployed on cloud servers for client users to visualize, manage, and share functional protein interaction networks.

**Results:**

With this R package, users can easily integrate downloaded protein-protein interaction information from different online databases with private data to construct new and personalized interaction networks. Additional functions allow users to generate specific networks based on private databases. Since the results produced with the use of this package are in the form of web pages, cloud users can easily view and edit the network graphs via the browser, using a mouse or touch screen, without the need to download them to a local computer. This package can also be installed and run on a local desktop computer. Depending on user preference, results can be publicized or shared by uploading to a web server or cloud driver, allowing other users to directly access results via a web browser.

**Conclusions:**

This package can be installed and run on a variety of platforms. Since all network views are shown in web pages, such package is particularly useful for cloud users. The easy installation and operation is an attractive quality for R beginners and users with no previous experience with cloud services.

## Background

The ability to visualize and manage protein-protein interaction (PPI) networks is extremely useful in biochemistry and other related research fields. Several online web servers are currently being used for these purposes. For example, the STRING database [1,2] not only provides PPI information for many organisms, but also graphically shows interactions of interest. The PINA database [3] is another online web server containing PPI information for six organisms and is also highly useful in this regard [4]. Both of these systems allow users to download PPI information they have collected from databases. However, users often experience difficulties in editing such collected information, as well as in constructing and sharing integrated PPI networks. The aim of this cisPath package is to simplify all of these processes. With cisPath, users can combine PPI information downloaded from different databases, add private data to these combinations, and construct new and personalized PPI networks. The functions available in this package are listed in Table 1.

**Table 1:** Functions of this cisPath Package.

Name	Description
formatPINAPPI	Format PPI file downloaded from PINA database
formatiRefIndex	Format PPI file downloaded from iRefIndex database
formatSTRINGPPI	Format PPI file downloaded from STRING database
combinePPI	Combine PPI information generated from different databases
getMappingFile	Generate protein identifier mapping file
networkView	Visualize input proteins in PPI network and display evidence that supports specific interactions among network proteins
cisPath	Identify and visualize shortest PPI paths between PPI network proteins
easyEditor	Open network graph editor

In recent years, cloud applications in which application software and all associated data are hosted centrally on the cloud have become more and more popular [5-7]. Cloud users typically access the software and data via a thin client web browser. RStudio [8] which was developed by RStudio Inc. and is available under a free software license for academic use is a powerful integrated development environment for R programming language. Deploying RStudio and R on a cloud server allows primary users to provide R as a cloud application to authorized users, who can access this R workspace from any computer. In this case, only primary users need to install and update R and related packages. Using R as a cloud application provides many benefits, but the necessity of downloading output to a local machine is an inconvenience that diminishes the benefit of the cloud model. Taking this into consideration, cisPath displays all of the results in web format. As such, using this package as a cloud application allows users to visualize, manage, and share results through the web browser, instead of downloading them. For users who may have only cloud drivers, such as Google Driver instead of cloud computing servers, results can be uploaded to cloud drivers, which can then be visualized and shared via a browser. Details on how to use Google Driver in hosting online results can be found on our website [9].

## Implementation

### Environment and technologies

R is a software environment that can be run on a variety of UNIX platforms, as well as Windows and Mac OS. This package employs R to analyze input files and output results in the HyperText Markup Language (HTML) format. In HTML files, PPI networks are displayed as Force-Directed Graphs with JavaScript library D3. To maintain consistency across different browsers, HTML files follow the HTML 4.01 Strict standard, HTML 5 experimental standard, and CSS version 3 standard. Thus, different browsers such as Chrome, Firefox, Safari, Opera and IE10, consistently display the output.

This cisPath package is available through the Bioconductor Project [10]. For users of cloud servers, RStudio is recommended as it enables primary users to provide a browser-based interface of a version of R running on a remote Linux server. Louis Aslett has provided various kinds of Amazon Machine Images (AMIs) which make deploying an RStudio Server very fast and easy [11]. These AMIs are highly recommended, especially for free micro instance users. The Bioconductor team has also developed AMIs optimized for running Bioconductor packages with the Amazon Elastic Compute Cloud (EC2) [12]. Users without any cloud services experience can easily launch the AMI using instructions on the Bioconductor website. An introduction on the use of this package for R beginners is also available on our website [9].

### Data collection and integration

There are several protein interaction databases, such as PINA, STRING, and iRefIndex [13,14], which allow downloading PPI information for academic purposes free of charge, but such downloaded files from different databases do not take on a common format. In this cisPath package, functions are provided to format the downloaded files from the PINA, STRING and iRefIndex databases into a standard workable format. To remove redundant interactions, UniProt Knowledgebase (UniProtKB) accession numbers are used as unique protein identifiers. UniProtKB is a part of the UniProt database and serves as a central hub for collection of functional information on proteins with accurate annotation [15]. UniProtKB consists of two sections including UniProtKB/Swiss-Prot (reviewed and manually annotated) and UniProtKB/TrEMBL (unreviewed and automatically annotated). Proteins with names that cannot be mapped to UniProtKB accession numbers are discarded.

The PINA database includes unified PPI data integrated from six manually curated databases: IntAct [16], MINT [17], BioGRID [18], DIP [19], HPRD [20] and MIPS MPact [21]. Like PINA, the iRefIndex database also provides an index of protein interactions integrated from primary interaction databases. PPI data downloaded from the PINA and iRefIndex databases contain the PubMed IDs of corresponding papers which support the PPIs. The STRING database contains not only known PPIs but also predicted protein associations with confidence scores. The latest version of STRING (v9.1) currently covers 5,214,234 proteins from 1,133 organisms. Although the PINA and iRefIndex databases are both integrated from manually curated databases, many distinct interactions exist in each case. Thus, several functions have been included in this package to format downloaded PPI data from different databases, consequently allowing users to edit downloaded information or merge them with privately collected data to construct more comprehensive PPI networks.

### Functions for visualization

**Figure 1:**
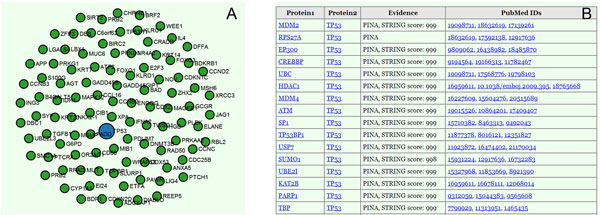
**Screenshots of the *networkView *function outputs**. (A) Visualization of the protein *TP53 *and interacting proteins. (B) Evidence supporting the specific interactions among these proteins.

After obtaining PPI data which has been downloaded from our website or privately generated, users can look up all the possible interactions for a given protein using the *networkView *function. Upon providing a gene name or the Swiss-Prot number for a given protein, proteins that are capable of interacting with the input protein are presented as shown in Figure 1A. The given protein is represented by a relatively larger blue node, while related proteins are presented as smaller green nodes (Figure 1A). These nodes can be clicked, which provide links to the UniProt database for the extraction of more information. Protein interactions are also presented in a table (Figure 1B), where the names of databases that support each specific interaction are displayed. PubMed IDs of corresponding publications and/or STRING scores are also displayed, providing users with direct links for the verification of corresponding sources as desired. In cases where there are more than 100 protein interactions for a given protein, the viewer randomly displays 100 of these interactions (Figure 1A), but all protein interactions and corresponding details are presented in the table (Figure 1B).

**Figure 2:**
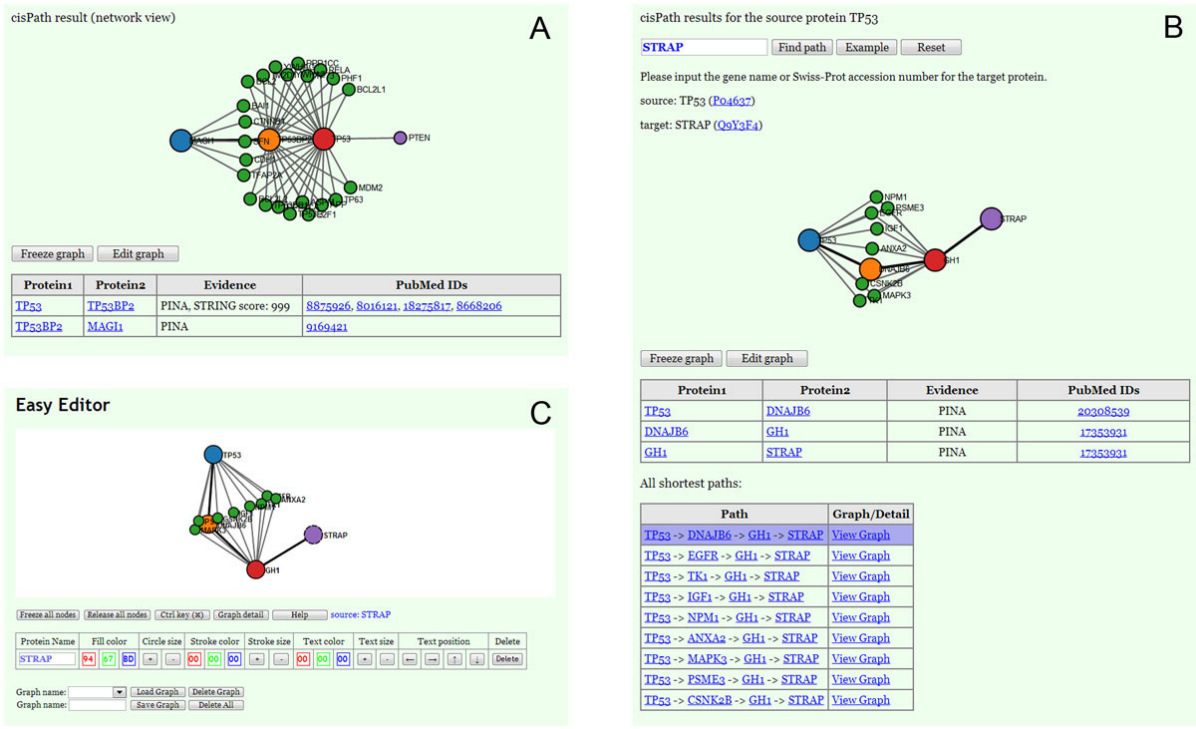
**Screenshots of the *cisPath *function outputs and network graph editor**. (A) PPI network visualization of the proteins *TP53*, *TP53BP2*, *MAGI1*, and *PTEN*. (B) Shortest interaction paths between proteins *TP53 *and *STRAP*. (C) Network graph editor.

The *networkView *function can also be used to visualize PPI networks in a given list of proteins, together with corresponding evidence of the specific interactions among them. A sample output is shown in Figure 2A, where the selected proteins are *TP53*, *TP53BP2*, *MAGI1*, and *PTEN*. The first three proteins are designated as main nodes and *PTEN *is designated as a leaf node. Since interaction between two proteins is often mediated by scaffolding proteins rather than direct interaction, the viewer also displays proteins that can interact with at least two of the main proteins, such as the green leaf nodes in Figure 2A. Additionally, users are free to choose the visualization style (color and node size) with which the proteins are displayed in the network. Using the parameter *mainNode *for this function, a selected protein can be designated as a main or leaf node. In this example, since PTEN is manually designated as a leaf node unlike the other three input proteins designated as main nodes, the only interactions presented for *PTEN *are those with the main nodes. Thus, views are generated corresponding to user preference. A protein often has multiple gene names, some of which may not be included in the input PPI data file. To avoid inputting invalid names of proteins, the unique identifier Swiss-Prot accession number may be used alternatively as input. Swiss-Prot accession numbers may be found in the UniProt database.

In some cases, users may want to identify interaction paths with more than two interacting steps between a pair of given proteins in a PPI network, and another function may be used to yield this type of result. The function *cisPath *identifies and outputs the shortest PPI paths between a pair of given proteins involved in multiple interaction steps. Users can obtain the shortest path(s) by either directly requesting the path(s) that reflect minimal cost using the default "cost" values of edges, or manually assigning "costs" to specific edges in the PPI network by editing the input file. The "cost" of an edge between two interacting proteins is a numerical value that is greater or equal to one, quantifying the extent to which an interaction is unfavorable. The default value for the "cost" of each edge generated from the PINA and iRefIndex databases is 1, and the "cost" of the edge generated from the STRING database is given as *max(1,log_100_^1000-STRING_SCORE^)*. The variable *STRING_SCORE *is the confidence score given by the STRING database. An example of this function is shown in Figure 2B. Evidence representing the STRING score or PubMed ID of relevant manuscripts is shown for all interaction paths. Similar to the *networkView *function, other proteins that can interact with at least two of the proteins that lie on the shortest PPI path are also displayed, giving a full range of possibilities despite the fact that they may be suboptimal paths. All of the shortest paths are listed in a table under the network view and can be shown graphically when selected (Figure 2B). To identify the paths that reflect the least number of steps independent of what the associated "costs" are, the parameter *byStep *may be set as TRUE. In this case, all edge "costs" are assigned as 1 and PPI paths with the minimum number of steps between a pair of given proteins are produced.

Research groups that focus on specific proteins may require screening of the shortest interaction paths from a single fixed protein to all other proteins in the input database. In this case, only the source protein name should be inputted in the *cisPath *function. All proteins in the input database are scanned for the shortest interaction paths to the fixed protein, and all of the shortest PPI paths from the fixed protein to each of the relevant proteins are outputted. Upon finding a new protein of interest, users can query the shortest interaction paths to the fixed protein with a browser without launching R. Although more CPU time and space is required to compute this function and store the results, results can be easily placed on a cloud driver or web server for quick access over the Internet. Sample results for fixed source proteins *TP53 *and *PTEN *can be found on our website.

The functions *networkView *and *cisPath *described above allow users to change color and size of the nodes in the network view prior to running. There is an additional editor for easy modification of network graphs after running. Figure 2C shows a screenshot of this tool. This editor is accessed via an "Edit graph" button on the output webpage, and allows users to make changes to the output graph as well as draw new network graphs that are directed or undirected, using different edge and arrow styles. The editor is compatible across a range of different browsers. Since most commonly used browsers support the HTML5 Web Storage, users can store the network graph view and open it later using the same browser. An additional function of this editor allows the view graph to be converted into a span of text. As the text is reversible to an editable view graph, it is possible to share output graphs easily via email or online messenger. This editor is independently usable, and is included in the source package. It is also available on our website for online access or downloading for offline usage.

## Results and discussion

As examples to demonstrate the use of this package, integrated PPI data from the PINA, iRefIndex and STRING databases for six model species are available for downloading from our website. Users should cite the databases accordingly when using these files. When integrating interactions from different databases, the function *combinePPI *will also count the number of valid proteins and protein interactions from each database. In view of the fact that the STRING database includes not only known PPIs but also predicted associations, only high confidence interactions from STRING are retained in these samples. The threshold of the STRING score is set to 700 by default, which can be changed manually with the function *formatSTRINGPPI*.

Taking Homo sapiens as an example, 14,646, 14,906, and 14,050 valid proteins are identified in the PINA, iRefIndex and STRING databases. Generated by the *combinePPI *function of this cisPath package, Tables 2 and 3 present the proteins and protein interactions in a given database which overlap with the other two databases. The last column of each table shows the number of distinct proteins or protein interactions found in each database. Although the PINA database contains the least number of distinct proteins, and has only 1008 proteins not found in the other two databases, it contains 36,867 interactions that are distinct, and most of these are supported by scientific literature. The iRefIndex database contains a large number of distinct proteins, as well as a large number of distinct interactions, which are all well supported by scientific literature. The STRING database contains up to 191,324 distinct interactions, but most of these interactions are predicted by data mining. Sample R scripts used to download and format PPI data can be found on our website. Only a small portion of available PPI data is selected for examples in the source package. The file with complete PPI information can be downloaded from our web site.

**Table 2:** Number of overlapping and distinct proteins listed for Homo sapiens in different databases.

Database	PINA	iRefIndex	STRING	Distinct proteins
PINA	14646	12539	11127	1008
iRefIndex	12539	14906	10518	1877
STRING	11127	10518	14050	2433

Each cell indicates the number of overlapping proteins from the two databases designated in the row and column; numbers on the diagonal indicate the total number of proteins from the database designated in the row and column. The last column indicates the number of distinct proteins from the given database.

**Table 3:** Number of overlapping and distinct protein interactions listed for Homo sapiens in different databases.

Database	PINA	iRefIndex	STRING	Distinct PPIs
PINA	106585	59500	26975	36867
iRefIndex	59500	132559	22564	67252
STRING	26975	22564	224106	191324

Each cell indicates the number of overlapping protein interactions from the two databases designated in the row and column, excluding PPIs with STRING scores of less than 700; numbers on the diagonal indicate the total number of proteins interactions from the database designated in the row and column. The last column indicates the number of distinct protein interactions from the given database.

Compared to other existing tools such as Cytoscape [22] and the online web servers PINA and STRING, this cisPath package is especially useful for R and cloud users. For R users, this package can be installed and updated by using only two commands, and no other plugins are required. Users can view, edit, and save the output network views with most modern web browsers. Cloud users can simply connect to the RStudio server with a browser and construct and publish personalized PPI databases and networks. It allows the user to play the role of PPI database administrator, rather than simply act as a common user.

## Conclusions

This cisPath package allows great convenience for cloud users who wish to visualize and manage functional protein interaction networks. With this package, PPI data from different databases can be integrated and used to deduce network graphs from one or several given proteins. This package can also identify the shortest interaction paths between a pair of proteins. Published evidence of interactions from different databases is present in the output HTML files. The graph editor adds a further layer of functionality, allowing different kinds of network views to be drawn according to user preference. With an RStudio server, molecular biologists can run cisPath functions and easily view results on mobile devices via web browsers. For cloud users, the Amazon EC2 service with AMIs which were generated by Louis Aslett is recommended [11]. One micro instance which is available free of cost is sufficient to launch the AMI and run this package.

In developing this cisPath package, one of our main aims is to offer an easy-to-use tool for mobile devices and panel PCs that is accessible through the browser. Therefore only functions that can be easily operated on mobile devices are included. A number of other functions, although useful, were excluded from the package, as they were deemed unsuitable for mobile use. We hope that with this tool, users will now be able to evaluate their ideas concerning protein interactions through visualization and management of protein networks, and limitations of time and location will become a thing of the past.

## Availability and requirements

Project name: cisPath

Project home page: http://www.isb.pku.edu.cn/cisPath/, http://www.bioconductor.org/

Availability: All sources and compiled code are free for academic use

Operating system(s): Platform independent

Programming language: R

Other requirements: R (>= 2.10.0)

License: GPL (>= 3)

Any restrictions to use by non-academics: None

## Competing interests

The authors declare that they have no competing interests regarding publication of this manuscript.

## Authors' contributions

LW developed the R package. LY, ZP, DL, YG, MM, and YY designed and tested its functions. All authors have read and approved the final manuscript.
